# Forensic Metabolomics: Enhancing PMI Estimation through Porcine Bone Tissue Profiling

**DOI:** 10.1021/acs.jproteome.5c00250

**Published:** 2025-10-15

**Authors:** Maria Elena Chiappetta, Elisa Roggia, Eugenio Alladio, Andrea Bonicelli, Noemi Procopio

**Affiliations:** 1 School of Law and Policing, Research Centre for Field Archaeology and Forensic Taphonomy, 6723 University of Lancashire, Preston PR1 2HE, United Kingdom; 2 Department of Biology Ecology and Earth Sciences, University of Calabria, Rende 87036, Italy; 3 Department of Chemistry, University of Turin, Turin 10124, Italy

**Keywords:** bone metabolomics, metabolites extraction, mass spectrometry, forensic sciences

## Abstract

The estimation of the post-mortem interval (PMI) in forensic skeletal remains is extremely challenging, as traditional morphological methods lose their effectiveness and accuracy as decomposition progresses. To address this issue, this study utilizes metabolomics to investigate the biochemical changes affecting bone tissue during the decomposition process. Fragments of pig mandibles were buried in an open grassland field at varying depths (0, 10, 30, and 50 cm) and collected every month up to 6 months. Bone metabolites were extracted using a single-phase methanol–water protocol, and both gas chromatography–mass spectrometry (GC-MS) and liquid chromatography-tandem mass spectrometry (LC-MS/MS) were applied for their analysis. The primary goal of this study is to identify specific metabolic shifts associated with increasing post-mortem intervals to identify potential bone metabolomic biomarkers for PMI and to assess the impact of burial depth on these changes. The generated regression models using LC-MS/MS data were able to estimate the PMI of the skeletal fragments with an accuracy of 14 days over 6 months, an outstanding result, particularly considering the current lack of methodologies to estimate PMI from bones. Burial depth, instead, did not play a significant role on the metabolomic bone signature. This research deepens our understanding of post-mortem biochemical processes in bones, making a significant contribution to the advancing field of forensic metabolomics, and highlights the potential of metabolomics for investigating buried skeletal remains and enhancing post-mortem interval assessments.

## Graphical abstract

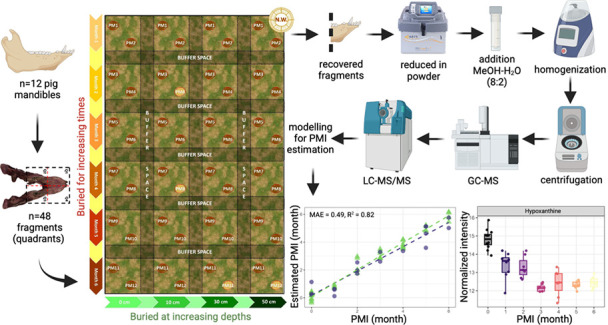

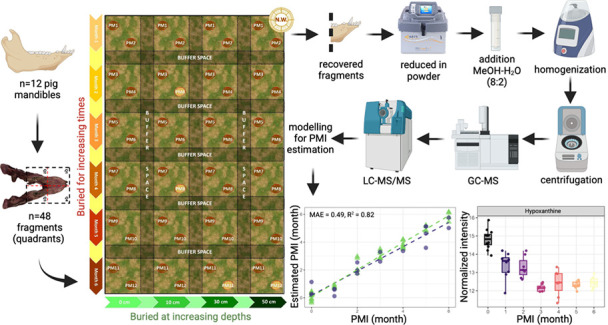

## Introduction

Estimating the post-mortem interval (PMI), or time since death, represents one of the most challenging tasks in forensic investigations dealing with deceased individuals. Traditionally, a variety of methods, including temperature-based approaches, and physical indicators such as post-mortem rigidity (rigor mortis) and cadaveric hypostasis (livor mortis), alongside transformative phenomena of the body and insect activity, are commonly employed for PMI estimation.^1–5^ However, these methods often face inherent limitations^6^ and become less effective or lose their applicability when applied to skeletonized remains, where PMI estimation becomes increasingly complex and less accurate. In these cases, forensic anthropologists rely on the morphological analysis of bones to estimate PMI. In the early stages of skeletonization, structures such as tendons and ligaments may persist, offering valuable clues for PMI estimation.^6^ However, the preservation of these soft tissues is heavily influenced by environmental conditions, as well as factors like exposure to scavengers, protective coverings (such as clothing or other materials), and burial depth.^7^ When exposed on the surface, skeletal remains undergo a process known as *bone weathering*, characterized by desiccation, bleaching, cortical surface exfoliation, and demineralization. This process, first systematically described by Behrensmeyer,^8^ has become a key tool for forensic anthropologists to assess PMI. Bone weathering progresses through distinct stages, from the absence of visible cracks or exfoliation in the early phases to advanced deterioration where the bone’s outer surface becomes rough, fibrous, and fragile, often leading to the exposure of trabecular bone.^8,9^ Despite its usefulness, bone weathering is not without limitations. The progression of bone deterioration can vary significantly depending on environmental conditions, making it challenging to standardize the process across different settings. Consequently, forensic anthropological analysis largely depends on the examiner’s experience and expertise, which introduces a risk of error and, in turn, can compromise the reliability and accuracy of the conclusions.

Several approaches based on physical and chemical principles have been developed to overcome the limitations inherent in bone weathering and other traditional methods to support PMI estimation in skeletonized remains. These include the application of UV fluorescence,^10,11^ IR and Raman spectroscopy,^12,13^ X-ray diffraction (XRD),^14,15^ the evaluation of citrate concentration,^16,17^ and the use of biomarkers such as hemoglobin,^18,19^ collagen,^20,21^ and DNA,^22^ whose degradation correlates with PMI. Recently, forensic investigations have increasingly turned to -omics technologies, due to their scientific robustness and ability to analyze complex molecular data, potentially providing a more precise and accurate estimation of PMI.

Several studies have explored metabolomics, the large-scale study of metabolites, across various tissues, demonstrating the potential of metabolic profiling to elucidate the complex biochemical changes that occur after death and thereby providing valuable information on the time since death. For example, Donaldson and Lamont^23^ employed untargeted metabolomics using gas chromatography–mass spectrometry (GC-MS) to analyze post-mortem blood in rats, identifying key metabolites, such as amino acids and citric acid cycle components, which exhibited time-dependent changes and showed potential to work as PMI biomarkers. Similarly, another study^24^ used liquid chromatography–mass spectrometry (LC-MS) to investigate blood samples in an in vitro animal model, focusing on the early post-mortem stage. This analysis identified hypoxanthine, lactic acid, histidine, and lysophosphatidic acids as significant metabolites that could serve as PMI indicators. Mora-Ortiz et al.^25^ demonstrated the utility of NMR-based metabolomics for detecting post-mortem biochemical changes, highlighting lactate, taurine, and niacinamide as promising markers for estimating the time of death. Expanding on this, Pesko^26^ used untargeted metabolomics to study both rat and human muscle tissues, identifying consistent time-dependent changes in amino acids like threonine and tyrosine, as well as small molecules such as xanthine, underscoring their reliability as PMI markers in forensic contexts. Du^27^ also contributed to this field by profiling rat femoral muscle at various post-mortem intervals using LC-MS, identifying metabolites such as N6-acetyl-l-lysine, nicotinamide, and inosine 5′-monophosphate, further emphasizing their potential as PMI biomarkers. More recently, Bonicelli^28^ explored a multiomics approach by integrating metabolomics, lipidomics, and proteomics in human bones for PMI estimation, demonstrating that combining these techniques could enhance estimation accuracy and precision.

This expanding field of research appears promising but also highlights the crucial role of animal models in forensic studies. While human cadavers offer direct relevance to forensic cases, their limited availability, their high interindividual variability, and the ethical constraints on experimental manipulations of human cadavers pose significant challenges. In contrast, animal models allow researchers to conduct controlled experiments with larger sample sizes, facilitating the repetition, replication, and validation of statistically robust results.^29^ Among the various species used, the domestic pig (*Sus scrofa*) is one of the most commonly employed in forensic research. Although pigs are not perfect analogues to humans, they share enough physiological similarities to offer valuable insights into forensic studies.^30^

The present study employs a metabolomic approach using GC-MS and liquid chromatography-tandem mass spectrometry (LC-MS/MS) to analyze pre- and postdecomposition pig mandible samples collected from 12 individuals, with the aim of investigating the biochemical changes associated with decomposition over a 6-month period and their relation to different burial depths (0, 10, 30, and 50 cm) to find potential metabolomic biomarkers for bone PMI estimation and to deepen our understanding of post-mortem biochemical processes in bones.

## Materials and Methods

### Sample Collection and Sub-Sample Preparation

This study obtained ethical approval from both the University of Northumbria Ethics Committee (ref. 11623) and the University of Central Lancashire Ethics Committee (ref. SCIENCE 0223).

Defleshed pig mandibles (*N* = 12) were purchased from a local butcher and were considered ″fresh″ with a PMI effectively set to zero despite the animals’ actual time of death. The mandibles were kept refrigerated at +4 °C for 1 day before the experiment began. Each mandible was sectioned into four quadrants by performing both longitudinal and transversal cuts (Figure 1) using a manual saw to limit bone molecular decay due to heating, producing four fragments that could be considered as biological replicates from a molecular point of view. To confirm the suitability of these fragments as replicates, preliminary proteomics and metabolomics tests were conducted on a separate test mandible from the same supplier. The results (data not shown) confirmed the biomolecular profile similarity among the four fragments, indicating no significant differences between each mandible portion.

**Figure 1:**
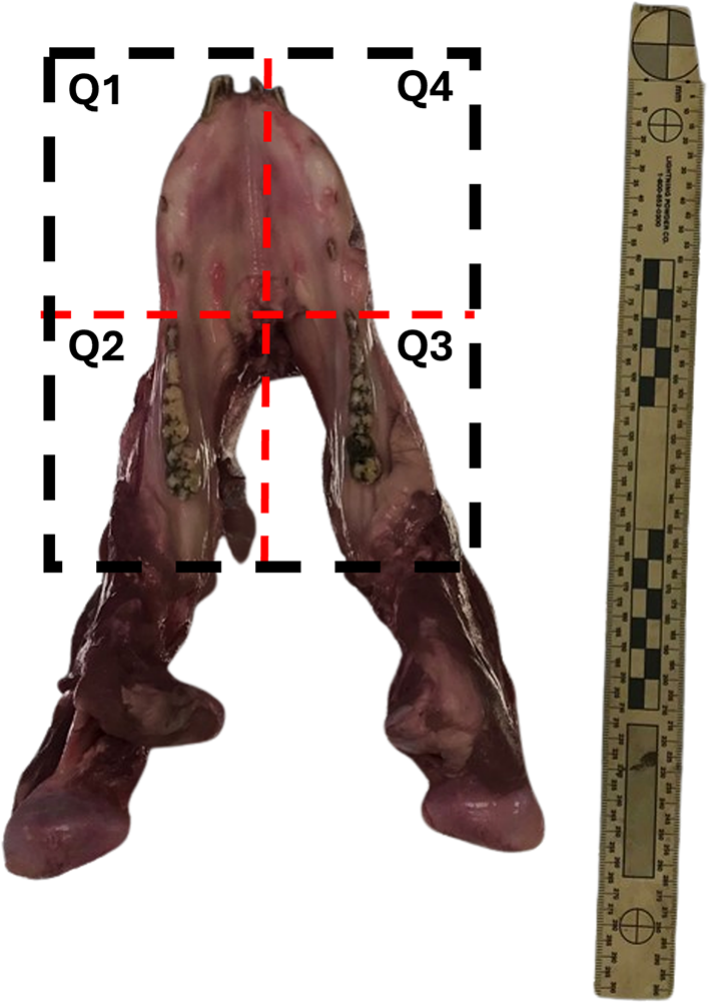
Sectioning of the pig mandible into four quadrants (Q1, Q2, Q3, and Q4).

The taphonomy experiment was conducted at the Taphonomic Research in Anthropology: Centre for Experimental Studies (TRACES) research site at the University of Lancashire. A 7.7 × 4.3 m area of grassland at 290 m altitude was selected for the experiment. This area had never been used for taphonomic experiments before and is located at the highest point in the research site, minimizing the risk of soil contamination from the percolation and flowing of decomposition fluids.

The burial depths selected were 0 cm (surface), 10, 30, and 50 cm, to simulate forensic scenarios where shallow burials tend to be most frequently encountered, with post-mortem intervals ranging from zero to six months. The plot was divided into smaller 70 cm^2^ squares, each designated for a specific soil depth and post-mortem interval. Specifically, moving from south to north, squares were allocated to the surface experiments, followed by those at 10, 30, and 50 cm depths, respectively. Moving from west to east, squares were designated for post-mortem intervals from one to six months (Figure 2). After digging holes to the required depth, the removed soil was replaced to cover the bone completely (except for surface experiments). Marking flags indicated the location of each buried quadrant were used. A 70 cm buffer zone was maintained between squares in the west to east direction and a 50 cm buffer zone in the south to north direction, to minimize potential soil contamination considering the area’s expected water flow direction (Figure 2). The entire area was covered with cages to prevent scavenger interference as much as possible.

**Figure 2:**
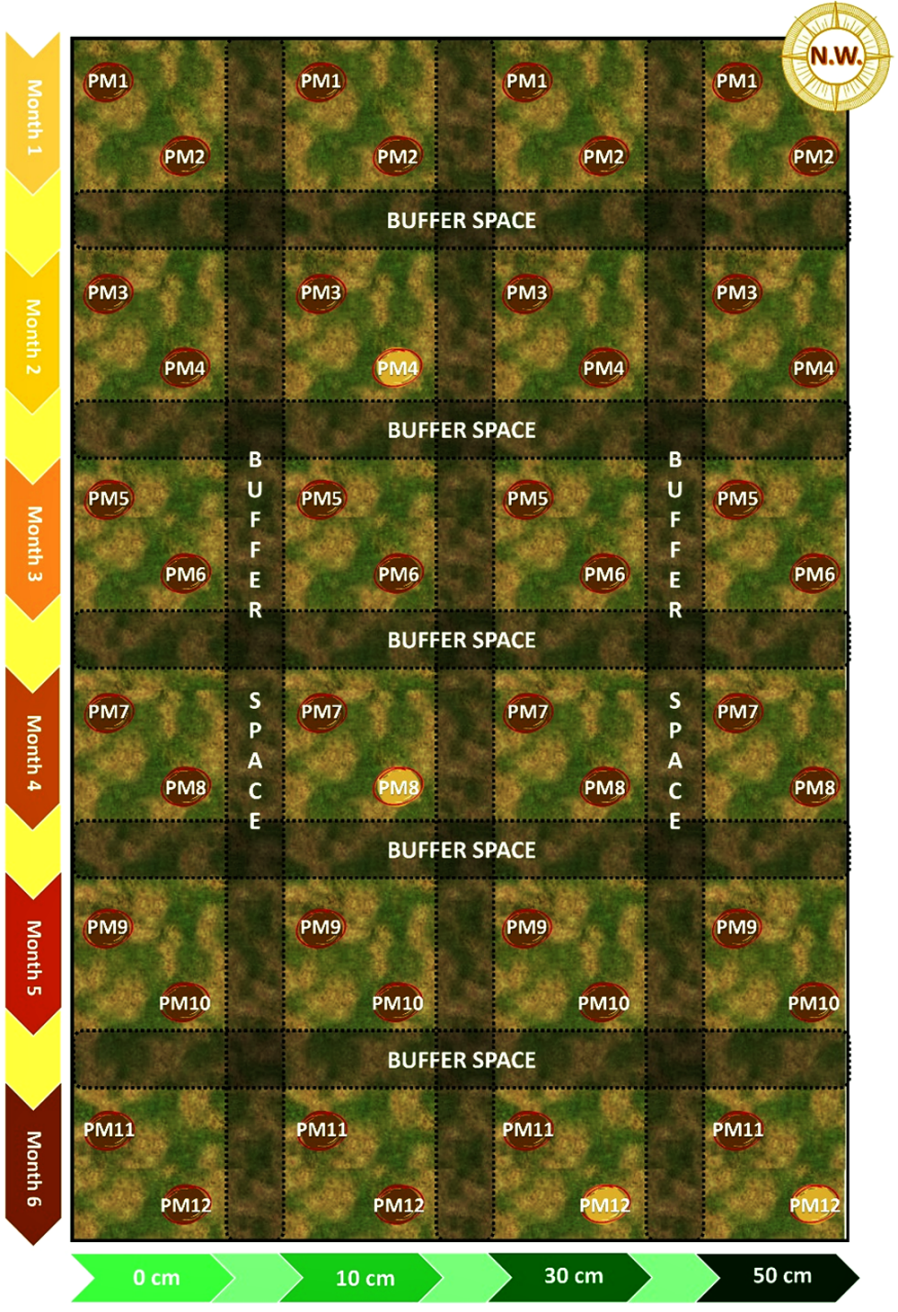
Experimental design of the taphonomic study; highlighted samples were not retrieved.

For each mandible, the four quadrants were placed in different squares at the selected depths (e.g., a mandible from one pig was used for PMI = one month, distributed across 4 different depths). Additionally, two distinct bone fragments from different mandibles were used to have two replicates for each test, and they were placed at opposite corners of each square to maximize their distance (Figure 2 and Table 1).

**Table 1:** Experimental Design of the Taphonomic Study, Including Samples Used in the Study, Burial Depth, and PMI

square number	pigs used per square	pig mandible (PM) code	burial depth	post-mortem interval
1	pig 1 and pig 2	PM1 – PM2	0 cm	one month
2	pig 3 and pig 4	PM3 – PM4	0 cm	two months
3	pig 5 and pig 6	PM5 – PM6	0 cm	three months
4	pig 7 and pig 8	PM7 – PM8	0 cm	four months
5	pig 9 and pig 10	PM9 – PM10	0 cm	five months
6	pig 11 and pig 12	PM11 – PM12	0 cm	six months
7	pig 1 and pig 2	PM1 – PM2	10 cm	one month
8	pig 3 and pig 4	PM3 – PM4	10 cm	two months
9	pig 5 and pig 6	PM5 – PM6	10 cm	three months
10	pig 7 and pig 8	PM7 – PM8	10 cm	four months
11	pig 9 and pig 10	PM9 – PM10	10 cm	five months
12	pig 11 and pig 12	PM11 – PM12	10 cm	six months
13	pig 1 and pig 2	PM1 – PM2	30 cm	one month
14	pig 3 and pig 4	PM3 – PM4	30 cm	two months
15	pig 5 and pig 6	PM5 – PM6	30 cm	three months
16	pig 7 and pig 8	PM7 – PM8	30 cm	four months
17	pig 9 and pig 10	PM9 – PM10	30 cm	five months
18	pig 11 and pig 12	PM11 – PM12	30 cm	six months
19	pig 1 and pig 2	PM1 – PM2	50 cm	one month
20	pig 3 and pig 4	PM3 – PM4	50 cm	two months
21	pig 5 and pig 6	PM5 – PM6	50 cm	three months
22	pig 7 and pig 8	PM7 – PM8	50 cm	four months
23	pig 9 and pig 10	PM9 – PM10	50 cm	five months
24	pig 11 and pig 12	PM11 – PM12	50 cm	six months

Bone subsamples from each quadrant were taken by cutting a small bone fragment (approximately 0.5 cm^3^) using a manual saw. Control samples (time 0) were collected from each mandible at the start of the experiment (*n* = 12 control samples in total). Additional samples were collected from each quadrant at the end of each selected time point in the experiment. All samples were stored at −20 °C until the experiment’s completion, after which they underwent metabolomic extraction.

The quadrants from PM4 buried at 10 cm, from PM8 buried at 10 cm and from PM12 buried at 30 and 50 cm were not recovered, likely due to scavenging activity or for other unforeseen reasons and were therefore not available for subsequent -omics analyses (Figure 2).

### Metabolite Extraction Experimental Workflow

The small bone samples collected from each mandible (*n* = 56 including the 12 controls collected at time 0) were reduced in powder with a 6775 Freezer/Mill Cryogenic Grinder (SPEX Sample Prep, Metuchen, NJ) under the following conditions: samples were precooled for 2 min in liquid nitrogen before being subjected to two grinding cycles. Each cycle consisted of a 2 min run at a rate of 7 Hz (cps), with 2 min of intermediate cooling time. The samples were placed in appropriate milling vials and submerged in liquid nitrogen to ensure cryogenic temperatures throughout the process. After grinding, the resulting bone powder was carefully collected for subsequent analysis. The extraction followed the protocol by Bonicelli et al.^31^ with minimal amendments. Specifically, 50 mg of bone powder were added to 2 mL prefilled bead mill tube containing ceramic beads (1.4 mm in diameter), and 950 μL of an 8:2 (% v/v) methanol–water solution was added. The samples were vortexed for 30 s and then homogenized using a Precellys Evolution Touch Homogenizer with four 20-s bursts at 5854 g, with a 2 min pause between bursts. The homogenization tube was then centrifuged at 18,213 g at 4 °C for 10 min, and 900 μL of the supernatant was transferred to a new tube. An additional 750 μL of the methanol–water solution was added to the original tube, and the homogenization process was repeated. After centrifugation at 18,213 g at 4 °C for 10 min, another 700 μL of supernatant was transferred to the same collection tube. This combined extract was centrifuged once more at 18,213 g at 4 °C for 10 min, after which 1.2 mL of the supernatant was transferred to a fresh tube and dried under a nitrogen flow. The dried extracts were then stored at −80 °C until further testing.

### GC-MS Analysis

Bone extracts were resuspended in 50 μL of methoxamine hydrochloride solution in pyridine (Sigma-Aldrich: TS45950) and incubated in a Thermomixer (Thermo Scientific) at 60 °C with shaking at 400 rpm for 15 min. After incubation, the samples were removed from the Thermomixer and left to cool. Subsequently, 50 μL of BSTFA + 1% TMCS (Supelco: B-023) was added, and the mixture was incubated again at 60 °C with shaking at 400 rpm for another 15 min. Negative controls consisting of methoxamine hydrochloride solution and BSTFA alone were prepared as blanks.

Gas chromatography–mass spectrometry (GC-MS) was carried out using an Agilent Intuvo 9000 GC system, coupled with an Agilent 5977B MSD single quadrupole mass spectrometer and 7693A autosampler. The system was operated through Masshunter Workstation GC/MS Data Acquisition software V10.1.49.

A sample volume of 1 μL was injected using an Agilent 10 μL Gold Standard syringe into a split–splitless double taper ultrainert liner with deactivated quartz wool (Agilent: 5190–3983), heated to 250 °C. The carrier gas, 99.9995% pure helium, was maintained at a total constant flow rate of 19.1 mL/min with a septum purge flow of 3 mL/min. A split ratio of 10:1 was used, resulting in a split flow of 11 mL/min, with the gas saver set to 15 mL/min after 3 min. The Intuvo flow path was maintained at 250 °C. An Agilent DB5-MS GC column was utilized, measuring 30 m in length, with a diameter of 250 μm and a film thickness of 2.5 μm. The carrier gas flowed through the column at a 1.1 mL/min rate. The oven was first equilibrated at 60 °C for 1 min, then ramped up at 10 °C/min to a final temperature of 325 °C, which was held for 10 min. Each sample had a total run time of 37.5 min.

The MS transfer line, source, and quadrupole temperatures were set to 290 °C, 250 °C, and 150 °C, respectively. A solvent delay of 5 min was implemented before data acquisition to protect the electron ionization (EI) filament. Data was collected between 50–600 *m*/*z* with a scan speed of 1562 u/s and 2.7 scans/second, resulting in a cycle time of 375 ms.

Data preprocessing was performed using ‘erah’ library^32^ in R version 4.4.1 (2024–06–14). Minimum compound peak width was set at 2 s, minimum noise threshold at 500, and masses between 35:69, 73:75, 147:149 *m*/*z* were not considered for the deconvolution step. Alignment parameters were minimum spectral correlation value at 0.1 and maximum retention time distance at 3 s. Putative annotation was performed at level 2 using the Golm open-source database for GC-MS (GMD_20111121_VAR5_ALK_MSP)^33^ based on retention time and spectral matching.

### LC-MS/MS Analysis

LC-MS analysis was conducted using a Thermo-Fisher Ultimate 3000 HPLC system, which included an HPG-3400RS high-pressure gradient pump, a TCC 3000SD column compartment, and a WPS 3000 Autosampler, all coupled to a SCIEX 6600 TripleTOF Q-TOF mass spectrometer with a TurboV ion source. The system was operated using SCIEX Analyst 1.7.1, DCMS Link, and Chromeleon Xpress software.

Samples were reconstituted in 95:5 (% v/v) water/acetonitrile. A 5 μL sample volume was injected using a pulled loop onto a 5 μL sample loop, followed by a 150 μL postinjection needle wash. The injection cycle time was set to 1 min per sample.

Reverse phase liquid chromatography was used for the chromatographic separation of metabolites. The separation was achieved using a Thermo Accucore C18 column (2.1 × 150 mm with particle size of 2.6 μm), operating at 40 °C with a flow rate of 3 mL/min. The LC gradient consisted of a binary solvent system, namely solvent ‘A’ (0.1% formic acid in water) and solvent ‘B’ (0.1% formic acid in 98:2 acetonitrile/water). The chromatographic gradient was programmed as follows: 5% ‘B’ at T0 with a hold for 1 min, followed by a linear increase to 100% ‘B’ at 8 min, hold for 2 min, return to starting condition and hold for 4 min for column re-equilibration.

The mass spectrometer operated under the following source conditions: curtain gas pressure at 50 psi, temperature at 400 °C, ESI nebulizer gas pressure at 50 psi, heater gas pressure at 70 psi, and declustering potential at 80 V. The voltage applied for ESI+ was 5500 V.

Mass spectrometry data were acquired in a data-dependent (DDA) mode, with features selected for fragmentation based on the top 10 most intense ions having a charge state of 1–2 and a minimum threshold of 10 cps. Isotopes within 4 Da were excluded from the scan. The accumulation time per scan was set at 100 ms, with the TOF survey scan accumulating data for 250 ms. The total cycle time was 1.3 s. Collision energy was calculated using the formula CE (V) = 0.084 × *m*/*z* + 12, up to a maximum of 55 V. Acquired data were reviewed using PeakView 2.2 and then imported into Progenesis QI 2.4 for metabolomic analysis, where they were aligned, peak-picked, normalized to all compounds, and deconvoluted following standard Progenesis QI workflows. Peak picking parameters were set to automatic with default sensitivity and a minimum peak width of 0.1 min.

MSI level 2 annotations were assigned by matching accurate mass, MS-MS spectrum, and isotope distribution ratios of the acquired data against the NIST MS-MS metabolite library (version 1.0.5673.40082, Progenesis QI plugin). Additionally, retention times and accurate masses were compared against an in-house library of chemical standards using Progenesis QI, with a 0.5 min retention time tolerance. Metabolites with a score higher than 40 were considered acceptable. MSI level 1 identifications were made when both libraries were in agreement and the MS-MS spectra matched NIST library entries.

### Blank and Quality Controls

Extraction blanks were prepared by executing the extraction protocol without any bone material. Pooled quality control (QC) samples were created by combining 10 μL aliquots from each sample, excluding the blanks. The QC pool was subsequently vortexed, centrifuged at 21,000 g for 10 min at 4 °C, and 100 μL of the supernatant was transferred into low recovery vials. These QC vials were analyzed at regular intervals throughout the run to monitor and control for instrumental drifts.

### Data Processing and Statistical Analysis

Data processing and statistical analysis were performed using the ‘structToolbox’ library^34^ in R version 4.4.1 (2024–06–14), if not otherwise stated. Only features detected in at least 80% for GC-MS and 90% for LC-MS of the samples were kept. Those with a fold change of less than 25 for GC-MS and 20 for LC-MS relative to the blanks were excluded. Pooled quality control (QC) samples from the metabolomics study were used to monitor for potential instrumental drifts, and features with a relative standard deviation exceeding 30% for GC-MS and 10% for LC-MS compared to the QC samples were discarded. Features were analyzed using multivariate techniques, including principal component analysis (PCA), intensity heatmap with hierarchical clustering, and partial least-squares regression. For the partial least-squares regression (PLSR) analysis, the data set underwent stratified splitting based on post-mortem interval into training, 70% of the data set, and test sets (30%) to evaluate the model’s predictive capability. The ‘caret’ package^35^ was employed for machine learning. Subsequently, univariate analyses were conducted, specifically the Kruskal–Wallis test followed by Dunn’s pairwise test with significance set at α ≤ 0.05.

The details regarding the data processing, analysis, and visualization can be found on the GitHub repository at https://github.com/abonicell/metabolomics-mandible.

### GC-MS Results

Originally, 837 features were identified; after applying the blank filter 680 were retained, after the filter for the missing values 62 were retained, and finally after the QC RSD filter 48 metabolites were putatively annotated. PCA (Figure 3A) explained 37.9% of the total variance through its first two principal components (PC1 and PC2), revealing a clear separation between fresh and decomposed samples. The preburial control samples (zero months PMI) formed a well-defined cluster on the left side of the plot, indicating a high degree of similarity and clear separation from other groups. In contrast, samples with a 1-month PMI were distinctly positioned at the top of the plot, clearly separated from both the control samples and those with longer PMIs. Samples with PMIs ranging from two to six months displayed a more diffuse distribution, spreading diagonally from the center toward the lower right area of the plot. Notably, samples with a 2-month PMI tended to cluster closer to the 1-month group, while those with PMIs of three and four months gradually spread further downward and to the right, suggesting a progressive shift in sample characteristics with increasing PMI. Samples with 5- and 6-month PMIs were positioned further from the center and exhibited greater dispersion.

**Figure 3:**
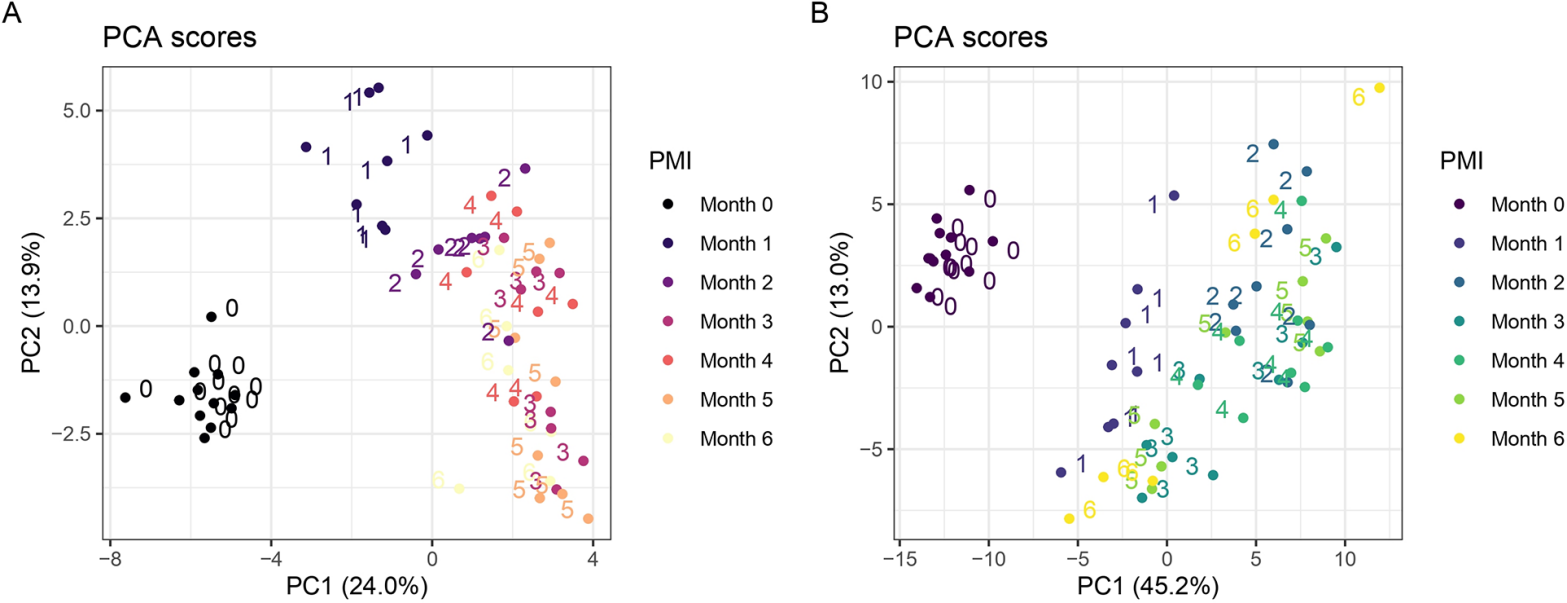
PCA of metabolites from pig mandible specimens at different PMIs. Plot (A) shows the results of GC-MS analysis, where PC1 and PC2 explain 24.0 and 13.9% of the total variance, respectively. The sample distribution varies with PMI, with noticeable clustering and dispersion patterns across time points. Plot (B) represents the LC-MS/MS analysis, where PC1 and PC2 explain 45.2 and 13.0% of the total variance, respectively. The grouping of samples reflects temporal trends across PMIs, with varying degrees of spread and one apparent outlier.

The hierarchical clustering analysis (Figure 4) supported the class separation observed in the PCA results, with distinct metabolic profiles for 0- and 1-month PMI samples, and increased homogeneity among longer PMIs. Regarding burial depth, no consistent pattern is observed, although samples buried at the same depth often cluster together. While many metabolic relative intensities tend to decrease with increasing depth, others exhibit variable behaviors, suggesting that depth alone does not predictably influence metabolic profiles.

**Figure 4:**
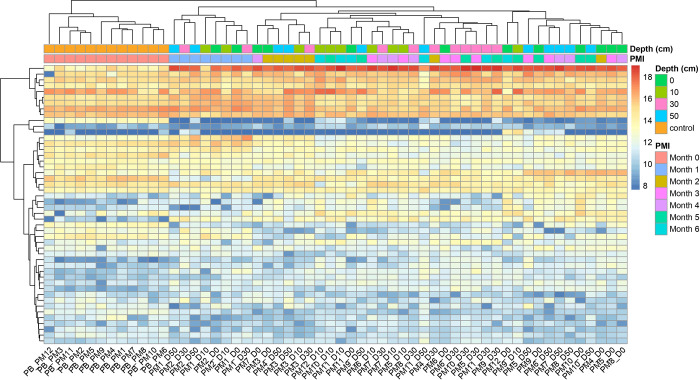
Hierarchical clustering heatmap of metabolic profiles by PMI and burial depth based on GC-MS data. The heatmap displays normalized metabolite intensities, with hierarchical clustering applied to both samples (columns) and metabolites (rows) to highlight patterns of similarity. Color bars above the heatmap indicate burial depth (top) and PMI (bottom), as defined in the legend. Warmer colors represent higher relative metabolite abundance, while cooler colors indicate lower abundance.

Since the obtained data suggested that the PMI had a more significant impact on metabolic profiles than the burial depth, a PLSR model was constructed using the metabolic patterns to estimate the PMI. The PLSR model plot (Figure 5A) illustrates the relationship between real PMI values and estimated PMI values for both the training and test sets. The model’s performance is quantified by the mean absolute error (MAE) of 0.82 months, indicating that, on average, the model’s prediction deviates from the actual values by approximately 24.6 days over a 6-month period. Coupled with an *R* ^2^ of 0.78, which reflects that the model explains 78% of the variance in the data, these metrics together demonstrate both good accuracy and predictive capability. Furthermore, the training and test samples follow a similar trend line, validating the model’s efficiency. Residual analysis was performed to further evaluate the GC-MS-based PLSR model (Figure S1A). Residuals were generally centered around zero, suggesting no major systematic errors, while slight variability was observed at higher PMI values. The overall distribution supports the model’s reliability in capturing PMI trends without significant bias.

**Figure 5:**
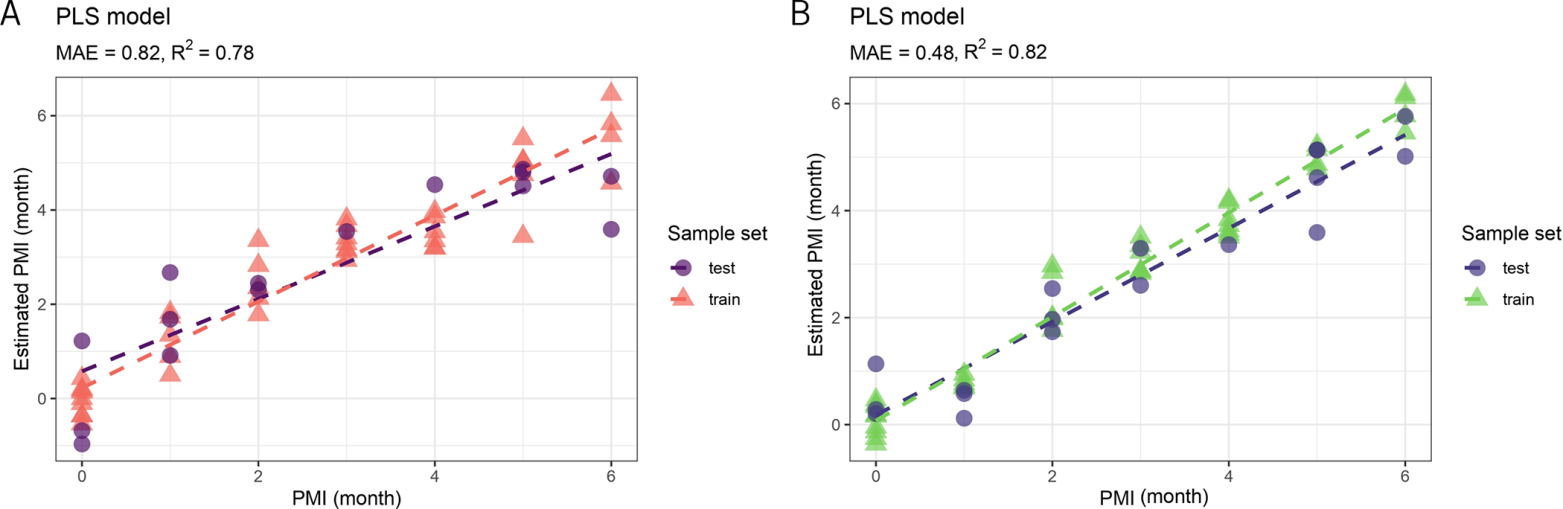
PLSR models for estimating PMI. Plot (A), based on GC-MS data, achieved an MAE of 0.82 months (approximately 24.6 days) and an *R* ^2^ of 0.78, demonstrating good predictive accuracy. Plot (B), using LC-MS/MS data, performed better with a lower MAE of 0.48 months (approximately 14.4 days) and an *R* ^2^ of 0.82, highlighting a more precise estimation of PMI across both training and test samples.

To further understand the impact of PMI on metabolites, a Kruskal–Wallis test was performed to compare metabolite intensities among different groups. The Kruskal–Wallis test identified several compounds as significant (Table S1). For instance, metabolites such as serotonin, hypoxanthine, myoinositol and N-acetylmuramate showed highly significant *p* values (*p* < 0.001), indicating substantial differences in their intensities across different PMI groups. However, these significant findings were not fully supported by Dunn’s pairwise test, which primarily revealed significant differences between a few specific PMI groups (Table S2), mainly between early and later PMIs. This suggests that the most pronounced metabolic changes occur early post-mortem, with fewer differences detected among later PMI groups. For example, hypoxanthine exhibited highly significant differences (*p* < 0.001) between PMI group zero and groups three, four, five and six. Similarly, ft625 (NA) showed highly significant differences (*p* < 0.001) between PMI group zero and groups three, four and five (Figure 6). Boxplots of metabolites showing significant differences are reported in Figure S2.

**Figure 6:**
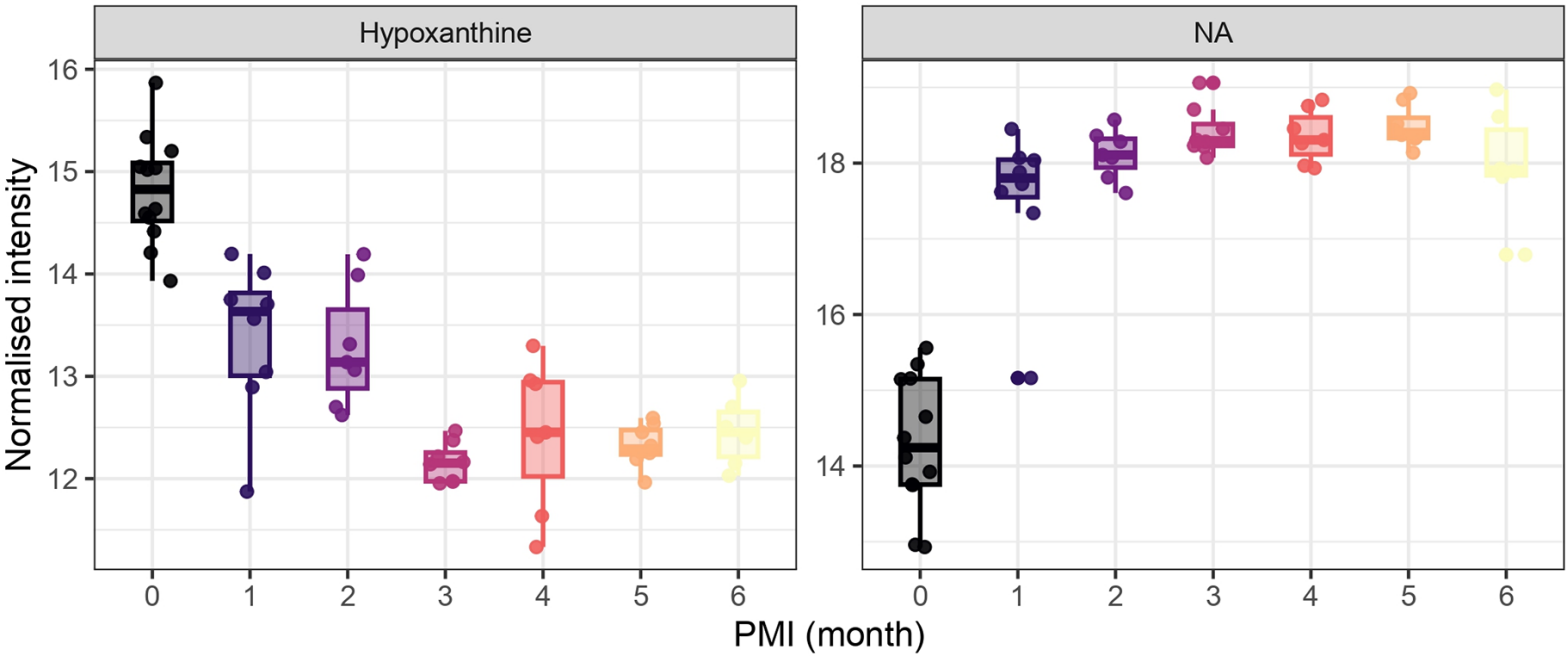
Boxplots of hypoxanthine and ft625 (NA) intensities across PMIs. Normalized intensities are plotted for each PMI group (0 to 6 months). Both metabolites show statistically significant differences between groups (*p* < 0.001), supporting their potential relevance as PMI-associated biomarkers.

The influence of burial depth on metabolite intensities was assessed using the same statistical approaches (Tables S3 and S4). The results show that most of the metabolites exhibit significant differences between the preburial and postburial conditions. However, only a few metabolites present significant variations between different depths, suggesting that while the overall burial environment impacts many metabolites, specific depth intervals have a more pronounced effect on a limited number of metabolites. This impact underscores the complexity of the decomposition process and the varying sensitivity of different metabolites to specific burial conditions.

### LC-MS/MS Results

Originally, 830 features were identified; after applying the blank filter 534 were retained, after the filter for the missing values 260 were retained, and finally after the QC RSD filter 147 metabolites were putatively annotated via MS/MS and retention time confirmation. The PCA results (Figure 3B) clearly distinguish the preburial control samples (zero months PMI) from the decomposed ones (1- to 6-month PMI). No significant interindividual differences can be observed among the control samples, as they formed a distinct cluster on the left side of the plot. In contrast, decomposed samples are more dispersed along the principal components PC1 and PC2, which explain 45.2% and 13.0% of the total variability, respectively.

Samples with a 1-month PMI tended to cluster along the first principal component, showing a degree of similarity and forming a distinct group. In contrast, samples with PMIs ranging from two to six months generally clustered together, suggesting a stabilization of characteristics over time. Despite this clustering, noticeable variability remained within this group, indicating that while differences became less pronounced, the samples were not completely uniform. Notably, an outlier in the upper right corner of the plot deviated significantly from the other samples; this deviation could be attributed to unique biological conditions, specific environmental factors, or data variability

The heatmap analysis (Figure 7), combined with hierarchical clustering based on PMI and burial depths, aligned with these findings, showing tighter clustering in 0- and 1-month samples, while later PMIs displayed greater dispersion. When examining the influence of burial depth on metabolic profiles, the results are somewhat inconclusive. Although samples buried at similar depths frequently clustered together, suggesting a potential link, no consistent pattern emerged across all data. While many metabolic relative intensities tended to decrease with increasing depth, others show variable behaviors, suggesting that the influence of burial depth is complex and not entirely predictable based on depth alone. Additionally, the distinct metabolic profile of the outlier identified in the PCA is clearly evident in the heatmap analysis.

**Figure 7:**
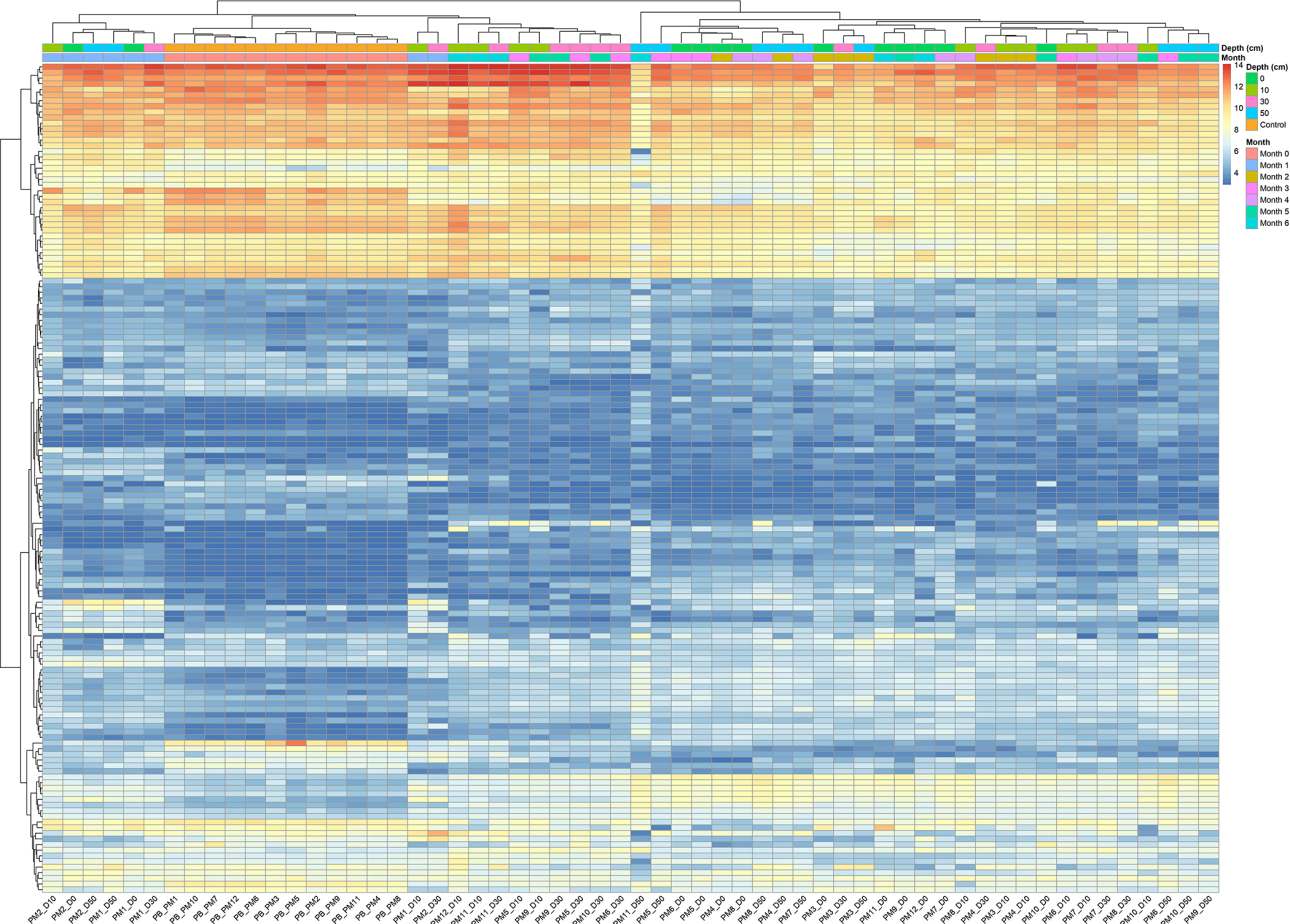
Hierarchical clustering heatmap of metabolic profiles by PMI and burial depth based on LC-MS/MS data. The heatmap visualizes relative metabolite abundances derived from normalized intensity data. Hierarchical clustering was applied along both axes to organize samples and metabolites based on similarity. Color-coded bars indicate sample metadata (burial depth and PMI). Color gradients reflect relative abundance, with warm colors indicating enrichment and cool colors indicating depletion.

The regression analysis using the PLSR model (Figure 5B) demonstrates a strong capability in predicting the post-mortem interval based on metabolic patterns, with a MAE of 0.48. This indicates that, on average, the model’s predictions deviate by about 0.48 months, or roughly 14.4 days, from the actual PMI over a 6-month period, reflecting a high level of accuracy. The *R* ^2^ value of 0.82 suggests that 82% of the variance in the data is explained by the model, highlighting its ability to capture the general relationship between actual and estimated PMI, despite some remaining variability. The results reveal a clear positive correlation: as the actual PMI increases, the estimated PMI correspondingly rises, indicating that the model effectively captures the underlying trend. The model’s strong performance is consistent across both the training and test sets, where the predictions closely align with the actual values. Residual analysis was conducted to further evaluate the LC-MS/MS-based PLSR model (Figure S1B). The residuals are evenly distributed around zero, with only minor variations, confirming the model’s robustness and accuracy in PMI estimation without evidence of systematic errors.

To gain a deeper understanding of the impact of PMI on metabolite levels, a Kruskal–Wallis test was conducted to compare metabolite intensities across different groups. The test revealed that 89.1% of the identified compounds exhibited statistically significant differences (Table S5). However, further analysis using Dunn’s pairwise test indicated that significant differences were primarily observed between fresh and decomposed samples, as well as among a few specific PMI groups, particularly between the first month and later intervals (Table S6). For example, *cis*-5,8,11,14,17-eicosapentaenoic acid exhibited significant differences (*p* < 0.05) between PMI group zero and groups two to six. Similarly, metabolite leukotriene D4 methyl ester showed significant differences (*p* < 0.05) between PMI group zero and groups two to six, as well as between PMI group one and groups two to five (Figure 8).

**Figure 8:**
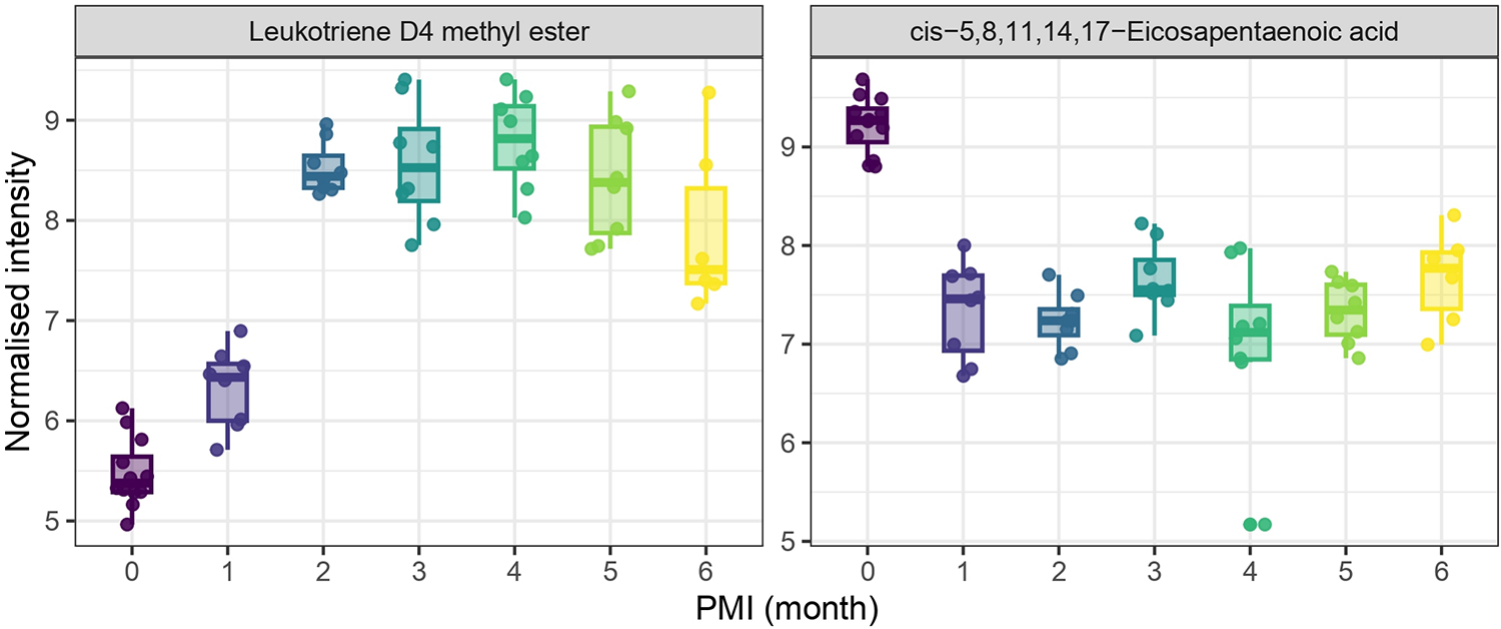
Boxplots of leukotriene D4 methyl ester and *cis*-5,8,11,14,17-eicosapentaenoic acid intensities across PMIs. Normalized metabolite intensities are shown for PMI groups ranging from 0 to 6 months. Both compounds display statistically significant differences between groups (*p* < 0.05), suggesting their potential association with PMI-related metabolic changes.

Regarding the burial depth, the results obtained using the same statistical approach indicate that most metabolites demonstrate significant differences when comparing preburial and postburial conditions (Tables S7 and S8). Nevertheless, only a limited subset of metabolites exhibits notable variations across different burial depths. This suggests that while the general burial environment substantially influences many metabolites, specific depth ranges significantly affect only a few. Boxplots illustrating the metabolites with significant differences are presented in Figure S3.

## Discussion

The analyses conducted using GC-MS and LC-MS/MS revealed distinct but complementary differences, providing an overview of the metabolic changes over time. Both techniques showed a clear separation between fresh and decomposed samples, but with some key differences in the nature and resolution of the metabolic information obtained.

The GC-MS analysis profiled 48 distinct metabolites, some of which significantly differentiated between fresh and longer PMI samples. The PCA (Figure 3A) highlighted these differences, with the tight clustering of preburial control samples indicative of consistent metabolic profiles and minimal interindividual variability prior to decomposition, and the distinct separation of 1-month PMI samples from both the control group and samples with longer PMIs, reflecting substantial changes in the metabolome within the first month after death. Beyond the first month, the metabolomic profiles of samples with PMIs ranging from two to six months displayed a more diffuse yet convergent distribution in the PCA, suggesting that after the first month, changes in the metabolome tend to converge, resulting in more similar metabolic characteristics among these samples. These results were supported by hierarchical clustering analysis (Figure 4), distinguishing the unique metabolic profiles of control and 1-month PMI samples from the more homogeneous characteristics observed in longer PMIs, reflecting a gradual reduction in differentiation as decomposition progresses.

In contrast, LC-MS/MS enabled the identification of a larger number of metabolites (147) compared to GC-MS, providing a more detailed overview of metabolic changes. Fresh samples were distinctly separated from buried ones, but PCA (Figure 3B) revealed greater dispersion among samples with PMIs between two and six months. This indicates that while significant metabolic changes occur within the first month post-mortem, the metabolome continues to evolve over the following months, eventually reaching a stabilization where differences become less pronounced, though some variability remains detectable. The PCA findings were also confirmed by the hierarchical clustering analysis (Figure 7). This ongoing variability is likely due to the ability of LC-MS/MS to detect a wider range of compounds, including polar, thermally labile and less volatile ones, which might not be captured by GC-MS. Consequently, LC-MS/MS may be better suited for detecting subtle and continuous metabolic changes that persist beyond the stabilization observed with GC-MS data.

Similarly, the better MAE observed in the PLSR regression model built on LC-MS/MS data compared to that using GC-MS data can be attributed to its greater sensitivity and resolution, which provided more detailed and accurate metabolic profiles, thereby enhancing the predictive capability of the model. Additionally, LC-MS/MS data often have better signal-to-noise ratios and fewer missing values, resulting in improved data quality that reduces noise and increases the reliability of the model’s input variables. This, combined with the larger number of predictors, allowed the PLSR regression model to better capture how the metabolome changes over time, leading to a more accurate representation of the relationship between metabolic profiles and PMIs,^36^ and resulting in a lower MAE of just 14.4 days over a period of six months.

Both analytical approaches highlighted that burial depth does not exert a consistent influence on metabolic profiles to mask the effect of PMI, although some significant differences were observed. GC-MS showed that while depth can affect the intensity of certain metabolites, there is no clear pattern associating greater depth with specific metabolic variations. This result might be attributed to environmental factors such as oxygen availability, microbial activity, and soil composition, which can vary independently of depth. Similarly, LC-MS/MS revealed that while most metabolites show significant differences between preburial and postburial conditions, only a minority of metabolites vary significantly across different burial depths. This suggests that while burial overall influences metabolism, specific burial conditions play a secondary role compared to the time elapsed since death.

The metabolic profiles of the GC-MS samples were predominantly characterized by lipids and their derivatives, such as cholesterol, linolenic acid, and arachidonic acid, alongside amino acids like valine and phenylalanine, and purine derivatives such as hypoxanthine. The high abundance of lipids and their derivatives reflects the breakdown of cellular membranes as cells disintegrate post-mortem due to the cessation of energy-dependent processes and subsequent enzymatic degradation. Additionally, some lipids, like elaidic acid, are associated with the animal’s diet,^37^ further influencing the metabolic profile observed in the samples. Similarly, the presence of alanine and other amino acids, such as phenylalanine, indicates the degradation of polypeptides and the subsequent release of amino acids following death.

Hypoxanthine, a well-known marker of ATP depletion and cellular energy crisis, accumulates after death when cells are deprived of oxygen or glucose. As ATP levels drop, adenine nucleotides are catabolized, with AMP (adenosine monophosphate) being converted to IMP (inosine monophosphate) and subsequently deaminated to produce hypoxanthine. The accumulation of hypoxanthine reflects the exhaustion of energy substrates and has been previously investigated for PMI estimation in vitreous humor^38,39^ and blood For instance, a recent study^40^ investigated post-mortem hypoxanthine levels in rat blood across varying ambient temperatures (5 °C, 15 °C, 25 °C, and 35 °C) over a 24 h period, reporting a consistent increase in hypoxanthine concentrations over time. In our study, hypoxanthine levels exhibited a marked increase during the initial months of decomposition, followed by a subsequent decrease and stabilization. This pattern may reflect the influence of environmental conditions and prolonged decomposition periods on hypoxanthine levels, extending its relevance to long-term PMI studies.

Additionally, several compounds were found to be associated with bacterial metabolism, such as N-acetylmuramate and 6-kestose. This observation aligns with existing knowledge that biomolecular decomposition is driven by a combination of enzymatic activity and microbial processes, ultimately leading to the production of bacterial metabolites.^28^ The results of the enrichment analysis performed on the GC-MS data are presented in Figure 9.

**Figure 9:**
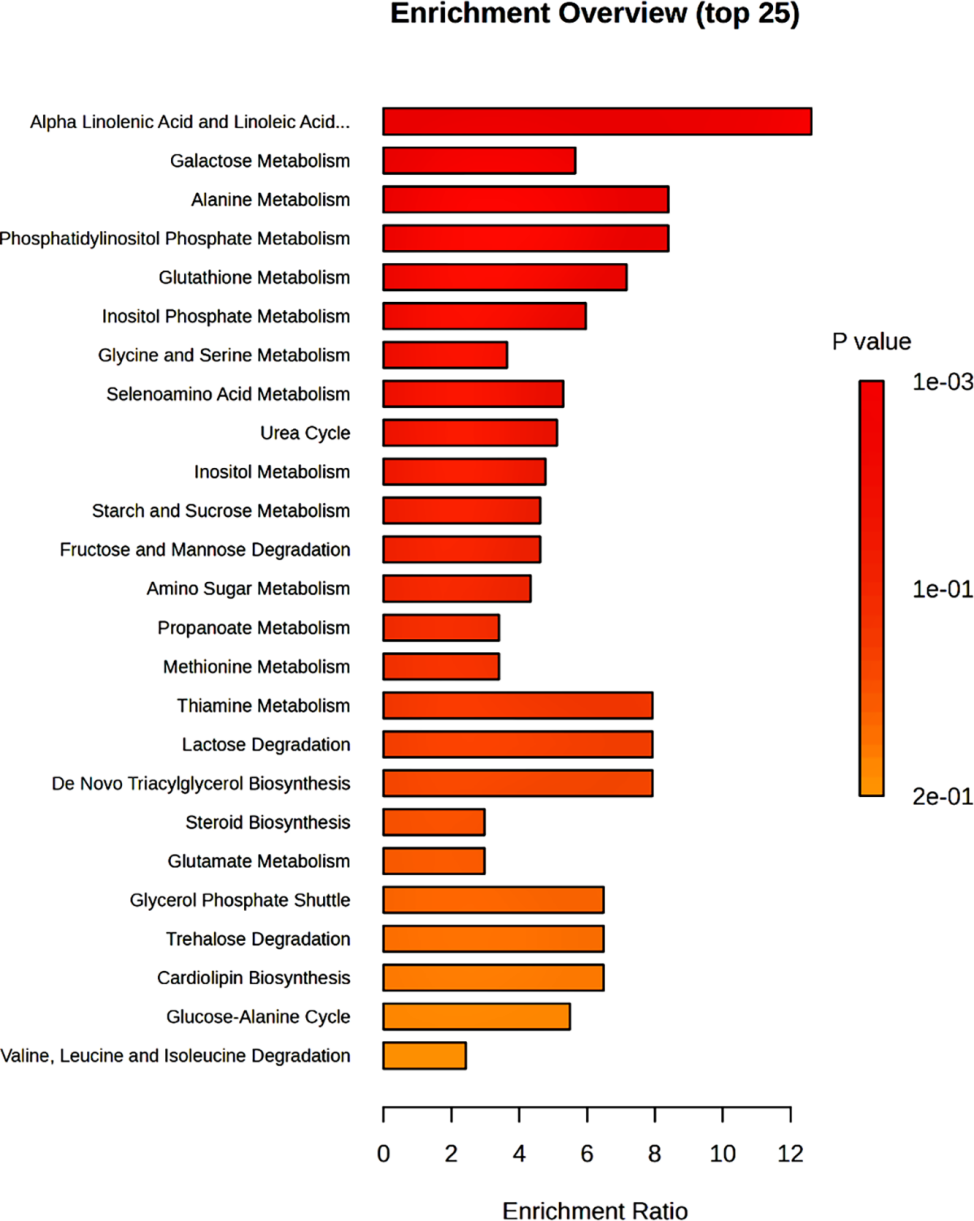
Enrichment analysis based on GC-MS data. The top 25 enriched metabolic pathways were identified from GC-MS-derived metabolite profiles using enrichment ratio and adjusted *p* values as ranking criteria. Pathways related to lipid metabolism (e.g., alpha-linolenic and linoleic acid metabolism), amino acid metabolism (e.g., alanine, valine, and methionine), and purine metabolism (e.g., hypoxanthine biosynthesis) were prominently enriched. The enrichment pattern reflects the biochemical changes occurring post-mortem, including lipid membrane degradation, protein breakdown, and energy depletion.

The LC-MS/MS data provided complementary insights, with metabolic profiles dominated by nucleic acid derivatives, including uracil, deoxyuridine, and xanthosine, peptides like prolylglycine and serylserine, and fatty acids. The prevalence of nucleic acid derivatives reflects the progressive degradation of DNA and RNA during tissue decomposition, which is further supported by the enrichment of pathways like pyrimidine metabolism (Figure 10). Notably, uracil has been previously studied as a marker for PMI estimation. For instance, an LC-MS study reported an increase in its levels within human muscle tissue across varying post-mortem intervals, with changes observed up to 14 days post-mortem in some cases.^26^ A similar pattern was observed in rat blood through GC-MS analysis.^41^ Consistent with these findings, Du^27^ also reported the presence of nucleic acid derivatives and specific metabolites, like N6-acetyl-l-lysine, in rat muscle tissues, highlighting their potential as PMI biomarkers. Peptides like prolylglycine and serylserine indicate ongoing protein breakdown, while fatty acids reflect the disintegration of cellular membranes providing further insights into the biochemical processes associated with tissue decay.

**Figure 10:**
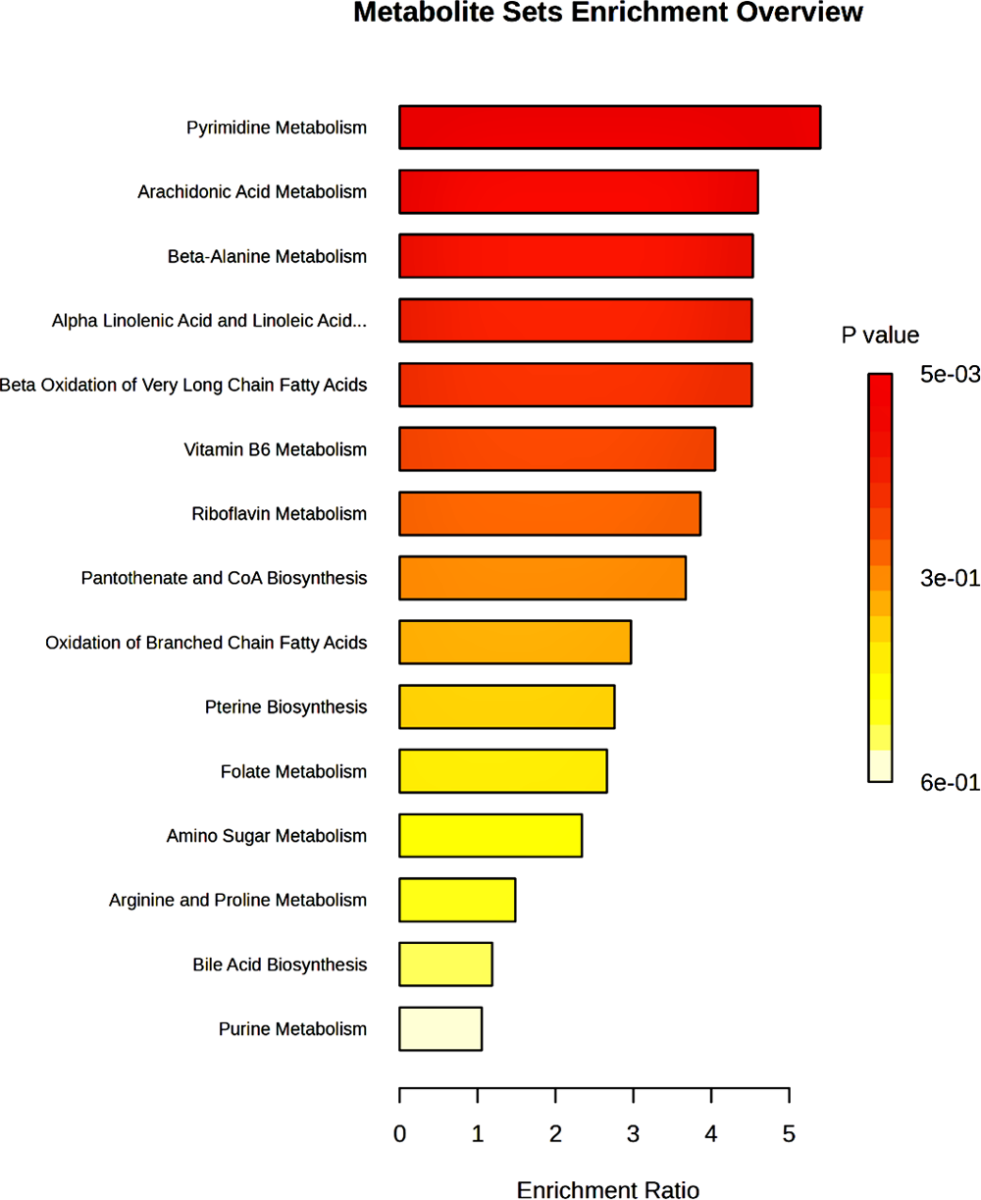
Enrichment analysis based on LC-MS/MS data. The plot shows the top enriched metabolic pathways ranked by enrichment ratio. Color intensity corresponds to adjusted *p* values, with darker shades indicating higher statistical significance. Pyrimidine metabolism, arachidonic acid metabolism, and beta-alanine metabolism are among the most enriched pathways, reflecting nucleic acid degradation, lipid metabolism, and protein breakdown occurring during post-mortem tissue decomposition.

Despite these promising results, several important limitations must be acknowledged that affect the interpretation and forensic applicability of our findings.

The use of porcine-derived tissue, while widely accepted in forensic taphonomy due to physiological similarities with human tissue,^30^ inherently limits the direct extrapolation of results to human remains. Although pigs serve as valuable analogues, species-specific differences in bone composition and post-mortem biochemistry may influence metabolite profiles, thereby requiring validation on human skeletal material before forensic implementation.

Another limitation relates to the environmental context of the study. The experiment was conducted under natural field conditions, but environmental variables such as temperature, humidity, precipitation, and soil pH, were not monitored directly on the site. Given that environmental parameters substantially influence decomposition dynamics and microbial activity,^42^ the absence of these data restricts in part our ability to contextualize the observed metabolic changes or assess the generalizability of the findings to other ecological settings.

Additionally, the loss of several bone samples, likely due to scavenging, reduced the number of replicates available for specific post-mortem intervals and burial depths. This limitation may have affected the statistical robustness of certain comparisons and the resolution of depth-related effects. More broadly, increasing the overall number of biological replicates would strengthen the statistical power of the analysis, enhance the reliability of the models, and improve the detection of metabolic trends associated with PMI and taphonomic variables.

The study also focused on a post-mortem interval of up to six months. While this time frame is representative of many forensic scenarios, it does not encompass longer decomposition periods that may be encountered in field cases. The applicability and predictive reliability of the models in such extended contexts remain to be determined.

Finally, while both gas chromatography–mass spectrometry and liquid chromatography-tandem mass spectrometry were employed, LC-MS/MS analysis was conducted exclusively in positive ionization mode. This choice may have limited the detection of metabolites preferentially ionized in negative mode. Future studies incorporating both positive and negative ionization would likely provide a more comprehensive metabolomic coverage, improving the sensitivity and reliability of biomarker identification for PMI estimation.

## Conclusions

The results of this study provide a better understanding of the impact of post-mortem decomposition on bone tissue metabolome, highlighting the potential of metabolomics as a powerful tool for estimating the PMI in forensic investigations. The ability to discriminate between different stages of decomposition using advanced metabolomic techniques represents a significant advancement over traditional methods. However, the variability observed in metabolic profiles suggests the need for further research to better understand the factors influencing metabolic decomposition. Validation of the models on a larger number of samples and under varying conditions will be essential to ensure the applicability to real forensic scenarios. In conclusion, both GC-MS and LC-MS/MS offer unique advantages for the study of post-mortem decomposition. GC-MS is particularly effective in analyzing volatile and thermally stable compounds, such as short-chain fatty acids and small organic molecules. On the other hand, LC-MS/MS is well-suited for detecting a broader range of metabolites, including larger, polar, and thermolabile molecules like nucleic acid derivatives and peptides. The combined use of both techniques offers a deeper and more accurate understanding of the post-mortem metabolic process, enabling the identification of critical metabolic pathways and biomarkers that would otherwise be challenging to detect with a single technique, while also improving the reliability of PMI estimation in forensic contexts.

## Supplementary Material





## Data Availability

All raw metabolomics data is available on Metabolomics Workbench: GCdata: https://dev.metabolomicsworkbench.org:22222/data/DRCCMetadata.php?Mode=Study&StudyID=ST003863&Access=LjzS9350, and LCdata: https://dev.metabolomicsworkbench.org:22222/data/DRCCMetadata.php?Mode=Study&StudyID=ST003862&Access=DloB9219

## Notes

Figure S1. Residual analysis of PLSR models for PMI estimation. Figure S2. Boxplots of selected GC-MS metabolites with significant variation across PMI groups. Figure S3. Boxplots of selected LC-MS/MS metabolites with significant variation across PMI groups. Table S1. Kruskal–Wallis test results on GC-MS metabolite intensities across PMI groups. Table S2. Dunn’s pairwise comparisons of GC-MS metabolite intensities across PMI groups. Table S3. Kruskal–Wallis test results on GC-MS metabolite intensities across burial depths. Table S4. Dunn’s pairwise comparisons of GC-MS metabolite intensities across burial depths. Table S5. Kruskal–Wallis test results on LC-MS/MS metabolite intensities across PMI groups. Table S6. Dunn’s pairwise comparisons of LC-MS/MS metabolite intensities across PMI groups. Table S7. Kruskal–Wallis test results on LC-MS/MS metabolite intensities across burial depths. Table S8. Dunn’s pairwise comparisons of LC-MS/MS metabolite intensities across burial depths (PDF)
Tables S1–S8 (XLSX)

## References

[1] Erbaş, M. Estimation of Death Time. In New Perspectives for Post-mortem Examination [Working Title]; IntechOpen, 2023, DOI: 10.5772/intechopen.1002056.

[2] Kori S. (2018). Time since
Death from Rigor Mortis: Forensic Prospective. J. Forensic Sci. Crim. Invest..

[3] Madea B., Kernbach-Wighton G. (2013). Early and
Late Postmortem Changes. Encycl. Forensic Sci..

[4] Laplace K., Baccino E., Peyron P. A. (2021). Estimation
of the time since death
based on body cooling: a comparative study of four temperature-based
methods. Int. J. Legal Med..

[5] Matuszewski S. (2021). Post-Mortem
Interval Estimation Based on Insect Evidence: Current Challenges. Insects.

[6] Hayman, J. ; Oxenham, M. Human Body Decomposition. Elsevier, 2016. doi: 10.1016/C2015-0-00038-7.

[7] Ubelaker, D. H. Postmortem Interval. In Encyclopedia of Forensic Sciences 24–27 Elsevier, 2013, DOI: 10.1016/B978-0-12-382165-2.00006-4.

[8] Behrensmeyer A. K. (1978). Taphonomic
and ecologic information from bone weathering. Paleobiology.

[9] Pokines, J. T. ; L’Abbé, E. N. ; Symes, S. A. Manual of Forensic Taphonomy. CRC Press: Boca Raton, 2021. doi: 10.4324/9781003171492.

[10] Sterzik V., Jung T., Jellinghaus K., Bohnert M. (2016). Estimating the postmortem
interval of human skeletal remains by analyzing their optical behavior. Int. J. Legal Med..

[11] Hoke N., Grigat A., Grupe G., Harbeck M. (2013). Reconsideration
of
bone postmortem interval estimation by UV-induced autofluorescence. Forensic Sci. Int..

[12] Wang Q. (2017). Estimation of the late postmortem interval using FTIR spectroscopy
and chemometrics in human skeletal remains. Forensic Sci. Int..

[13] Woess C. (2023). Raman spectroscopy for
postmortem interval estimation of human skeletal
remains: A scoping review. J. Biophotonics.

[14] Piga G., Malgosa A., Thompson T. J. U., Enzo S. (2008). A new calibration
of
the XRD technique for the study of archaeological burned human remains. J. Archaeol Sci..

[15] Rubio L., Suárez J., Martin-de-las-Heras S., Zapico S. C. (2023). Partners in Postmortem
Interval Estimation: X-ray Diffraction and Fourier Transform Spectroscopy. Int. J. Mol. Sci..

[16] Schwarcz H. P., Agur K., Jantz L. M. (2010). A New Method for Determination of
Postmortem Interval: Citrate Content of Bone*. J. Forensic Sci..

[17] Wilson S. J., Christensen A. M. (2017). A test
of the citrate method of PMI estimation from
skeletal remains. Forensic Sci. Int..

[18] Caudullo G. (2017). Luminol testing in detecting
modern human skeletal remains: a test
on different types of bone tissue and a caveat for PMI interpretation. Int. J. Legal Med..

[19] Ermida C., Cunha E., Ferreira M. T. (2024). Luminol and the
postmortem interval
estimation — influence of taphonomic factors. Int. J. Legal Med..

[20] Jellinghaus K., Hachmann C., Hoeland K., Bohnert M., Wittwer-Backofen U. (2018). Collagen degradation
as a possibility to determine the post-mortem interval (PMI) of animal
bones: a validation study referring to an original study of Boaks
et al. (2014). Int. J. Legal Med..

[21] Guareschi E. E., Pember L., Magni P. A. (2024). The degradation
of collagen in submerged
bones, analysed by ImageJ® and Orbit®, for the estimation
of the post-mortem interval (PMI) and the post-mortem submersion interval
(PMSI). Australian Journal of Forensic Sciences.

[22] Tozzo P., Scrivano S., Sanavio M., Caenazzo L. (2020). The Role of DNA Degradation
in the Estimation of Post-Mortem Interval: A Systematic Review of
the Current Literature. Int. J. Mol. Sci..

[23] Donaldson A. E., Lamont I. L. (2015). Metabolomics of
post-mortem blood: identifying potential
markers of post-mortem interval. Metabolomics.

[24] Szeremeta M. (2023). In Vitro Animal Model
for Estimating the Time since Death with Attention
to Early Postmortem Stage. Metabolites.

[25] Mora-Ortiz M., Trichard M., Oregioni A., Claus S. P. (2019). Thanatometabolomics:
introducing NMR-based metabolomics to identify metabolic biomarkers
of the time of death. Metabolomics.

[26] Pesko B. K. (2020). Postmortomics: The Potential of Untargeted
Metabolomics to Highlight
Markers for Time Since Death. OMICS.

[27] Du T. (2018). Metabolic profiling
of femoral muscle from rats at different periods
of time after death. PLoS One.

[28] Bonicelli A. (2022). The ‘ForensOMICS’
approach for postmortem interval
estimation from human bone by integrating metabolomics, lipidomics,
and proteomics. eLife.

[29] Simmons, T. ; Cross, P. A. Forensic Taphonomy. In Encyclopedia of Forensic Sciences 12–17 Elsevier, 2013. doi: 10.1016/B978-0-12-382165-2.00004-0.

[30] Matuszewski S. (2020). Pigs vs people: the
use of pigs as analogues for humans in forensic
entomology and taphonomy research. Int. J. Legal
Med..

[31] Bonicelli A., Taylor G., Procopio N. (2024). Extraction and untargeted analysis
of metabolome from undemineralised cortical bone matrix. Mol. Omics.

[32] Domingo-Almenara X. (2016). eRah: A Computational Tool Integrating Spectral Deconvolution and
Alignment with Quantification and Identification of Metabolites in
GC/MS-Based Metabolomics. Anal. Chem..

[33] Kopka J. (2005). GMD@CSB.DB: the Golm
Metabolome Database. Bioinformatics.

[34] Lloyd G. R., Jankevics A., Weber R. J. M. (2021). struct: an R/Bioconductor-based framework
for standardized metabolomics data analysis and beyond. Bioinformatics.

[35] Kuhn M. (2008). Building Predictive
Models in R Using the caret Package. J. Stat.
Software.

[36] Procopio N., Bonicelli A. (2024). From flesh
to bones: Multi-omics approaches in forensic
science. Proteomics.

[37] Liu H. (2023). Metabolomics Analysis Provides Novel Insights into the Difference
in Meat Quality between Different Pig Breeds. Foods.

[38] Lendoiro E. (2012). Applications of Tandem
Mass Spectrometry (LC–MSMS) in estimating
the post-mortem interval using the biochemistry of the vitreous humour. Forensic Sci. Int..

[39] Zelentsova E. A. (2020). Post-mortem changes
in metabolomic profiles of human serum, aqueous
humor and vitreous humor. Metabolomics.

[40] Fang S. (2024). A pilot study investigating early postmortem interval
of rats based
on ambient temperature and postmortem interval-related metabolites
in blood. Forensic Sci. Med. Pathol.

[41] Dai X. (2019). An experimental study
on investigating the postmortem interval in
dichlorvos poisoned rats by GC/MS-based metabolomics. Leg Med..

[42] Mason A. R., McKee-Zech H. S., Steadman D. W., DeBruyn J. M. (2024). Environmental predictors
impact microbial-based postmortem interval (PMI) estimation models
within human decomposition soils. PLoS One.

