# Supplementary material for: Forensic Metabolomics: Enhancing PMI Estimation through Porcine Bone Tissue Profiling
Source: J Proteome Res. 2025 Oct 15;24(11):5498–510. doi: 10.1021/acs.jproteome.5c00250 (PMC12604033; doi:10.1021/acs.jproteome.5c00250)
Supplement: Supplementary file 1 [file pr5c00250_si_001.pdf]

Supporting Information

Forensic Metabolomics: Enhancing PMI Estimation Through Bone Tissue Profiling

Maria Elena Chiappetta[1,2], Elisa Roggia[3], Eugenio Alladio[3], Andrea Bonicelli[1], Noemi Procopio[1*]

[1]School of Law and Policing, Research Centre for Field Archaeology and Forensic Taphonomy, University of Central Lancashire, Preston, United Kingdom; [2]Department of Biology Ecology and Earth Sciences, University of Calabria, Rende, Italy; [3]Department of Chemistry, University of Turin, Turin, Italy.

*****Correspondence: nprocopio@uclan.ac.uk; Tel.: +44 01772893493.

Table of Contents

Supplementary Figures

Figure S1 - Residual analysis of PLSR models for PMI estimation.

Figure S2 - Boxplots of selected GC-MS metabolites with significant variation across PMI groups.

Figure S3 - Boxplots of selected LC-MS/MS metabolites with significant variation across PMI groups.

Figure S1 - Residual analysis of PLSR models for PMI estimation.

(A) Residuals from the GC-MS-based PLSR model.

(B) Residuals from the LC-MS/MS-based PLSR model.

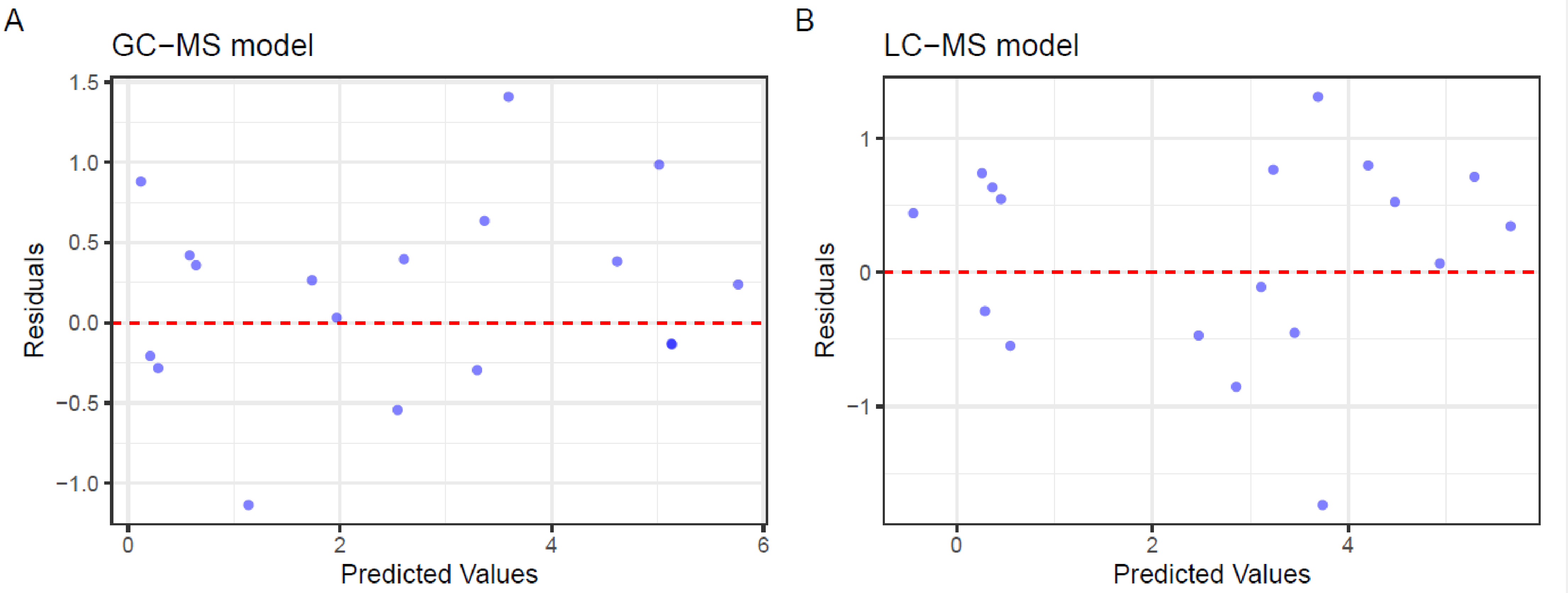

Figure S2 - Boxplots of selected GC-MS metabolites with significant variation across PMI groups.

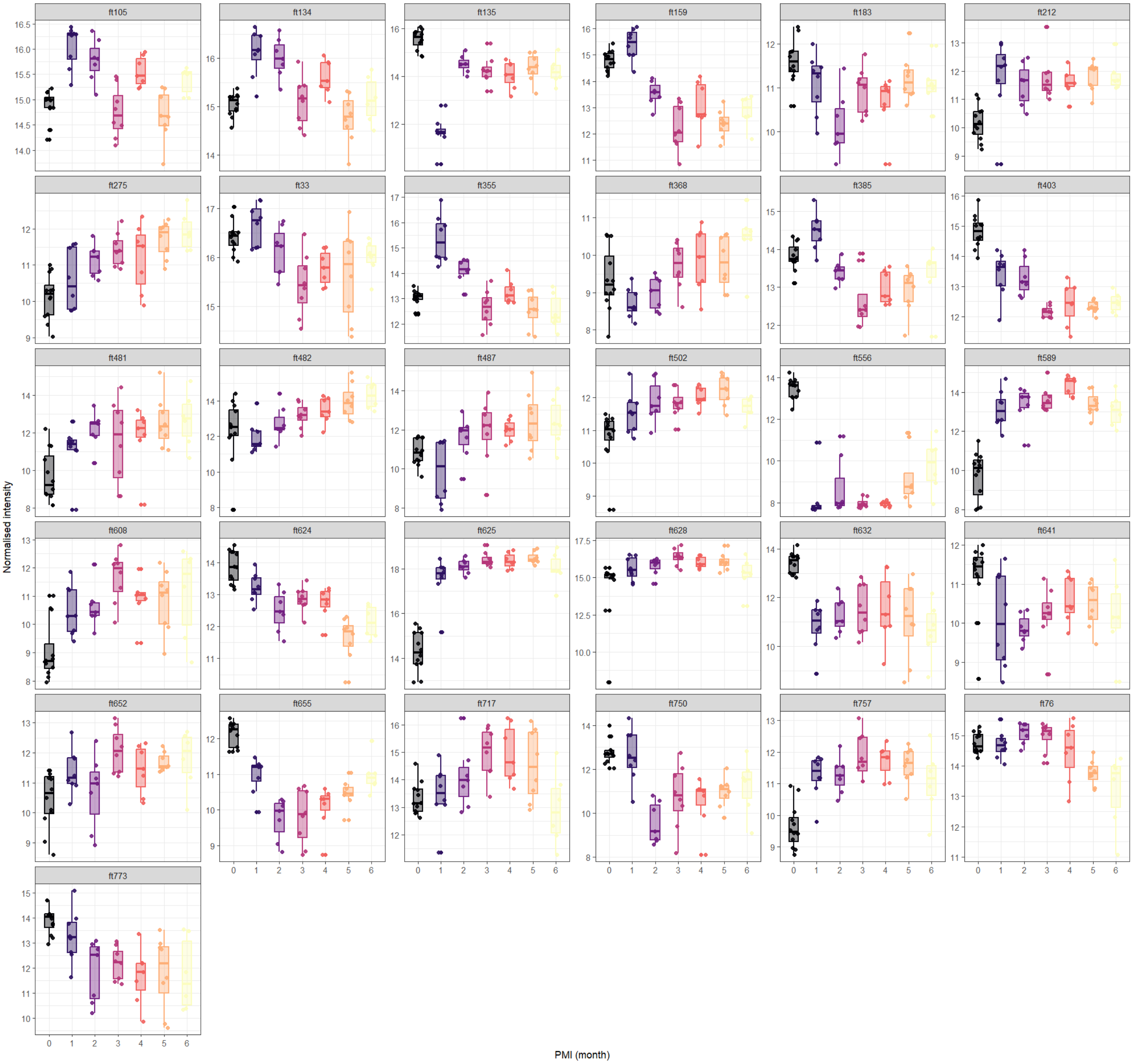

Figure S3 - Boxplots of selected LC-MS/MS metabolites with significant variation across PMI groups.

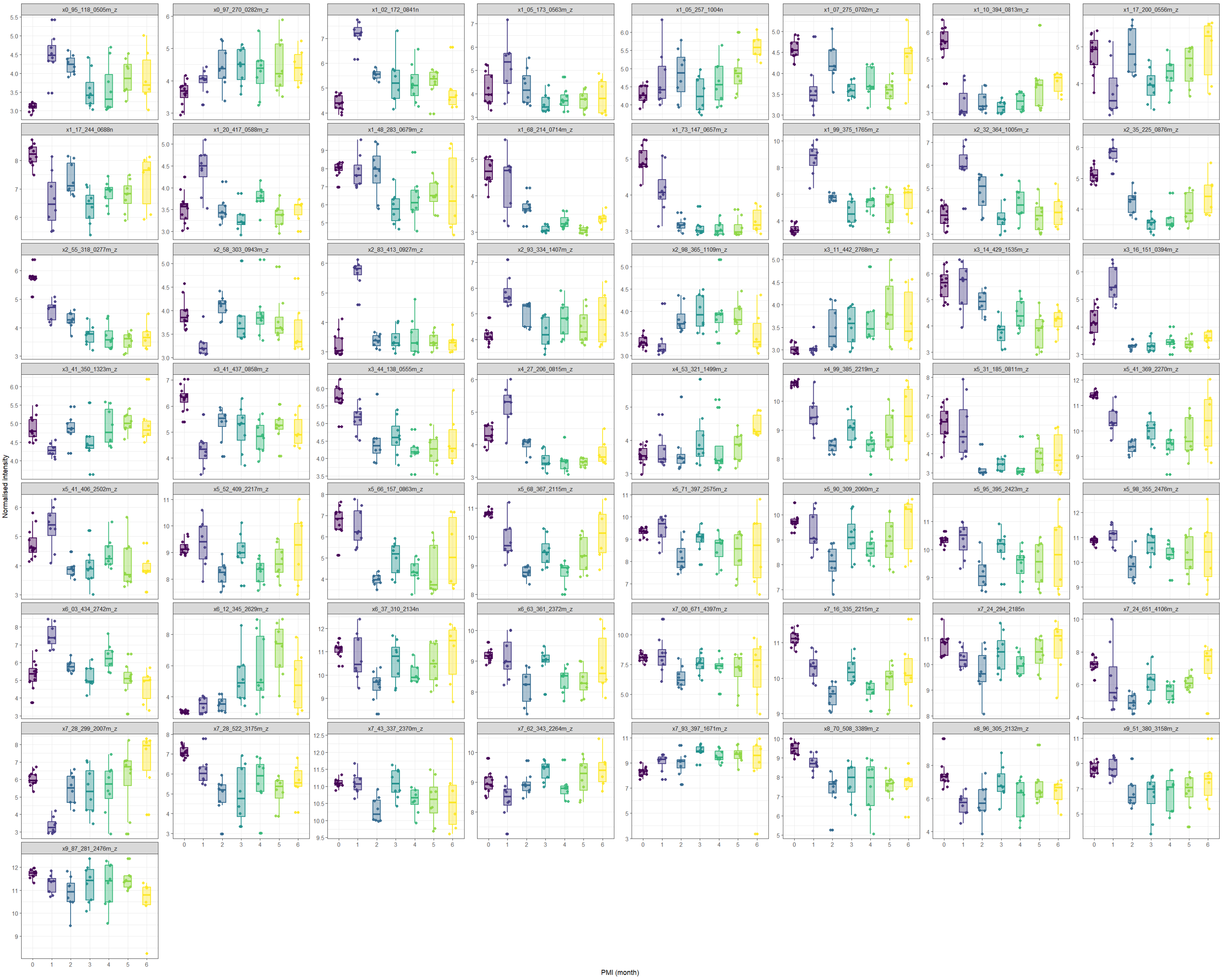

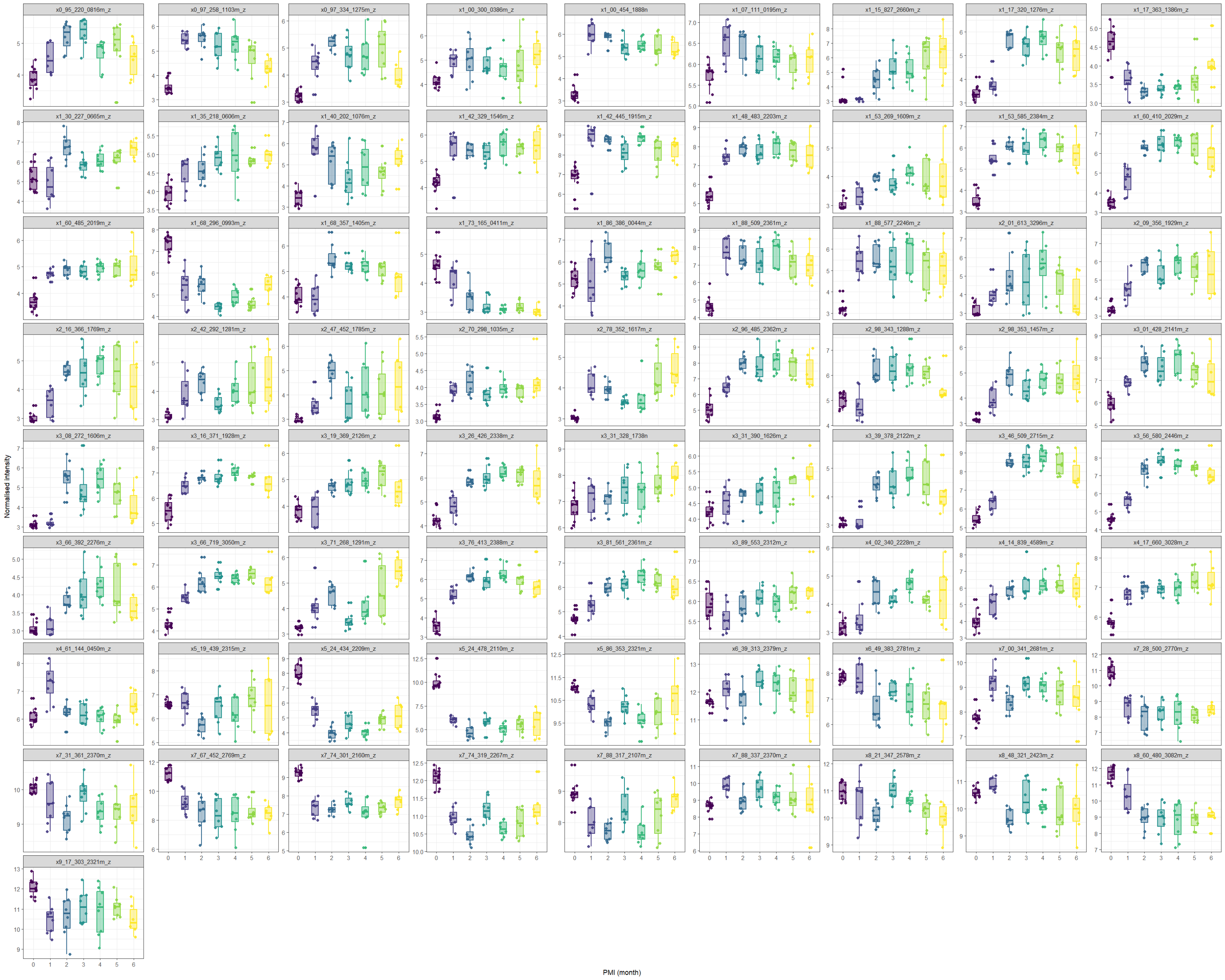
x0_95_220_0816m_z
x0_97_258_1103m_z
x0_97_334_1275m_z
x1_00_300_0386m_z
x1_00_454_1888n
x1_07_111_0195m_z
x1_15_827_2660m_z
x1_17_320_1276m_z
x1_17_363_1386m_z
x1_30_227_0665m_z
x1_35_218_0606m_z
x1_40_202_1076m_z
x1_42_329_1546m_z
x1_42_445_1915m_z
x1_48_483_2203m_z
x1_53_269_1609m_z
x1_53_585_2384m_z
x1_60_410_2029m_z
x1_60_485_2019m_z
x1_68_296_0993m_z
x1_68_357_1405m_z
x1_73_165_0411m_z
x1_86_386_0044m_z
x1_88_509_2361m_z
x1_88_577_2246m_z
x2_01_613_3296m_z
x2_09_356_1929m_z
x2_16_366_1769m_z
x2_42_292_1281m_z
x2_47_452_1785m_z
x2_70_298_1035m_z
x2_78_352_1617m_z
x2_96_485_2362m_z
x2_98_343_1288m_z
x2_98_353_1457m_z
x3_01_428_2141m_z
x3_08_272_1606m_z
x3_16_371_1928m_z
x3_19_369_2126m_z
x3_26_426_2338m_z
x3_31_328_1738n
x3_31_390_1626m_z
x3_39_378_2122m_z
x3_46_509_2715m_z
x3_56_580_2446m_z
x3_66_392_2276m_z
x3_66_719_3050m_z
x3_71_268_1291m_z
x3_76_413_2388m_z
x3_81_561_2361m_z
x3_89_553_2312m_z
x4_02_340_2228m_z
x4_14_839_4589m_z
x4_17_660_3028m_z
x4_61_144_0450m_z
x5_19_439_2315m_z
x5_24_434_2209m_z
x5_24_478_2110m_z
x5_86_353_2321m_z
x6_39_313_2379m_z
x6_49_383_2781m_z
x7_00_341_2681m_z
x7_28_500_2770m_z
x7_31_361_2370m_z
x7_67_452_2769m_z
x7_74_301_2160m_z
x7_74_319_2267m_z
x7_88_317_2107m_z
x7_88_337_2370m_z
x8_21_347_2578m_z
x8_48_321_2423m_z
x8_60_480_3082m_z
x9_17_303_2321m_z
Normalised intensity
PMI (month)
